# Universal nomogram for predicting referable diabetic retinopathy: a validated model for community and ophthalmic outpatient populations using easily accessible indicators

**DOI:** 10.3389/fendo.2025.1557166

**Published:** 2025-06-12

**Authors:** Niu Dongling, Kang Ziwei, Sun Juanling, Zhang Li, Wang Chang, Lei Ting, Liu Hongli, Zhang Yanchun

**Affiliations:** ^1^ Xi’an People’s Hospital (Xi’an Fourth Hospital), Affiliated People’s Hospital of Northwest University, Xi’an, Shaanxi, China; ^2^ Department of Fundus Surgery (Division IV), Shaanxi Eye Hospital, Xi’an, Shaanxi, China

**Keywords:** referable diabetic retinopathy, nomogram construction, community screening, risk stratification, easily accessible indicators

## Abstract

**Purpose:**

This study aimed to develop and validate a universal nomogram for predicting referable diabetic retinopathy (RDR) in type 2 diabetes mellitus (T2DM) patients, using easily accessible clinical indicators for both community and ophthalmic outpatient populations.

**Methods:**

A cross-sectional study was conducted with 1,830 T2DM patients from 14 communities in Xi’an, Shaanxi, China. Participants completed questionnaires, underwent physical exams, and ophthalmic assessments. Univariate analysis and least absolute shrinkage and selection operator (LASSO) regression identified key predictors for RDR. A nomogram was developed using multivariable logistic regression. Model performance was evaluated through area under the curve (AUC), accuracy, precision, recall, F1 score, Youden index, calibration curves, and decision curve analysis (DCA). The dataset was split into training (80%) and test (20%) sets, with external validation using 123 T2DM outpatients from Shaanxi Eye Hospital.

**Results:**

Seven key predictors were identified: serum creatinine, urea nitrogen, urine glucose, HbA1c, urinary microalbumin, diabetes duration, and systolic blood pressure. The nomogram exhibited moderate predictive accuracy, with AUCs of 0.730 (95% CI: 0.691–0.759), 0.767 (95% CI: 0.704–0.831), and 0.723 (95% CI: 0.610–0.835) for the training, test, and external validation sets, respectively. DCA showed that using the model is beneficial for threshold probabilities between 8% and 72%, supporting its broad clinical utility.

**Conclusion:**

This nomogram, based on readily available clinical indicators, provides a reliable and scalable tool for predicting RDR risk in both community and ophthalmic settings. It offers a practical solution for early detection and personalized management of RDR, with broad applicability and clinical potential.

## 1 Introduction

Diabetes mellitus (DM) has reached epidemic proportions, currently affecting over 460 million people worldwide and projected to exceed 700 million by 2045 (1, 2). Diabetic retinopathy (DR), one of the most serious microvascular complications of DM, remains the leading cause of irreversible vision impairment in working-age adults (3, 4). Clinical strategies for DR management emphasize three pillars: (1) systemic risk factor control, (2) population-based screening, and (3) targeted treatment for vision-threatening DR (vtDR). However, despite technological advances in teleophthalmology and artificial intelligence–assisted image analysis, current screening programs are constrained by their dependence on dilated fundus examination or retinal photography, both of which require trained personnel and specialized equipment (5). This resource dependency is particularly limiting in primary care settings, where the majority of DR cases are first encountered (6). Diagnostic challenge is most critical for referable diabetic retinopathy (RDR)-typically defined as more than mild nonproliferative DR (NPDR) and/or any stage with macular edema (ME) as defined by the the International Clinical Diabetic Retinopathy (ICDR), namely, any retinal thickening, exudate, or microaneurysm within 1 disc diameter of the fovea (7). In many regions, especially those with limited resources, a mismatch between screening infrastructure and patient needs leads to delayed diagnosis and suboptimal care. Studies have shown that up to 50% of RDR cases remain undetected until advanced stages (8, 9), and that early identification combined with standardized treatment can reduce the risk of blindness by up to 98% (10). Community-based DR screening has consistently demonstrated better cost-effectiveness and accessibility compared to managing late-stage complications, yet its scalability remains challenged by logistical and financial constraints.

In this context, predictive models have emerged as promising adjuncts to improve DR risk stratification and pre-screening efficiency. A variety of risk engines, ranging from simple clinical scoring systems to machine learning–based algorithms, have been proposed for DR prediction (11, 12). Among them, nomograms have gained prominence for their ability to synthesize multiple predictors into intuitive, individualized risk assessments, facilitating evidence-based clinical decision-making (13). Nonetheless, existing DR prediction models face several notable limitations. First, most models rely heavily on cumulative glycemic exposure (e.g., diabetes duration and HbA1c), which, according to large epidemiological datasets, accounts for only ~11% of the variance in DR risk (14). Second, many models incorporate specialized diagnostic parameters, such as optical coherence tomography (OCT), diabetic peripheral neuropathy assessments, or ankle-brachial index, limiting their applicability in primary care or low-resource settings (15–17). Third, generalizability is often undermined by methodological constraints: most models are developed and validated in single-center, hospital-based cohorts without external validation, which may introduce selection bias and reduce applicability to broader populations (15–21).

To address these gaps, we developed a simplified yet robust RDR risk prediction nomogram based solely on routinely collected clinical data—including blood pressure, glycemic status, renal function markers, and urine glucose—from a large-scale community-based cohort in Xi’an, China. The model was then externally validated in an independent tertiary outpatient population, enabling assessment of its generalizability across healthcare levels and demographic subgroups. By focusing specifically on RDR rather than any DR, the model directly supports clinical referral decisions, which is critical for preventing vision-threatening disease progression. This approach emphasizes clinical usability, implementation feasibility, and translational relevance, especially in primary care environments where access to ophthalmologic resources is limited. Ultimately, our nomogram offers a scalable and cost-effective tool for early risk identification, optimized resource allocation, and enhanced integration of DR screening into routine diabetes management.

## 2 Methods

### 2.1 Data source and collection

This cross-sectional study was conducted from 2021 to 2023 across 14 community health service centers in Shaanxi Province, China, and at the ophthalmology outpatient department of Xi’an People’s Hospital (Xi’an Fourth Hospital). The inclusion criteria were: adults (age ≥ 18 years) with type 2 diabetes mellitus (T2DM), diagnosed according to the World Health Organization (22). All participants must have been permanent residents of the selected communities and listed in the community chronic disease registries. They should have undergone diabetic retinopathy screening during the study period. Finally, they must have had access to key clinical and laboratory data for analysis. The exclusion criteria were as follows: patients who were younger than 18 years, those diagnosed with type 1 or gestational diabetes, individuals with insufficient data regarding diabetes duration or other key variables, pregnant or lactating women, and patients with malignancies or severe systemic illnesses that could impact ocular status or data completeness (e.g., end-stage renal disease on dialysis). Additionally, patients with ocular co-morbidities such as severe cataracts, retinal vein occlusion, or advanced age-related macular degeneration were excluded, as these conditions could confound the assessment of diabetic retinopathy (DR). Patients who met the criteria were recruited consecutively, and comprehensive evaluations were conducted using a standardized questionnaire and examination protocol. The study adhered to the Declaration of Helsinki and was approved by the Ethics Committee of Xi’an People’s Hospital (Xi’an Fourth Hospital). All participants provided written informed consent prior to enrollment.

Demographic factors (age, sex), lifestyle factors (smoking and alcohol use, exercise habits), socioeconomic status (education level, occupation, income), and medical history (duration of diabetes, medications, comorbid conditions) were recorded. All measurements were performed by trained healthcare staff. Physical examinations included blood pressure measurements using an electronic sphygmomanometer; anthropometric measurements (height, weight, waist and hip circumferences) for body mass index (BMI) and waist-to-hip ratio calculation. These candidate predictors were selected based on known DR risk factors in the literature and their availability in routine practice (18).

Subsequent to a minimum fasting period of 8 hours, fasting blood and urine samples were collected in the morning. fasting blood and urine samples were obtained in the morning for laboratory analyses. Blood tests included fasting plasma glucose, glycated hemoglobin (HbA1c), and a full biochemical panel: total cholesterol, triglycerides, high-density lipoprotein cholesterol, low-density lipoprotein cholesterol, blood urea nitrogen (BUN), serum creatinine (Scr), uric acid, and estimated glomerular filtration rate (eGFR). A complete blood count was also done (white blood cell count and differential, red blood cell count, hemoglobin, hematocrit, etc.), from which the neutrophil-to-lymphocyte ratio (NLR) was derived. Urine tests included a standard urinalysis (which detects qualitative urine glucose and protein) and measurement of urinary microalbumin (mALB) or albumin-to-creatinine ratio if indicated. All laboratory assays were performed in the hospital’s clinical laboratory center using standardized methods and quality control. These candidate variables were selected based on known risk factors for DR reported in the literature and their availability in routine clinical practice. By focusing on commonly measured indicators, we aimed to maximize the model’s practicality in general healthcare settings.

Visual acuity was measured and intraocular pressure (IOP) was checked using a non-contact tonometer (Icare ic100, Icare Finland Oy, Finland). Slit-lamp biomicroscopy (KJ5S1, Suzhou Kangjie Medical Inc, China) was performed to assess the anterior segment. For fundus evaluation, we obtained digital fundus color photographs for each eye using the Horus DEC 200 device (Miis, Hsinchu, Taiwan), employing a 45-degree double field centered on the fovea and optic disc, with or without pharmacologic pupil dilation. Two experienced retinal specialists, masked to clinical data, independently diagnosed and graded DR by the fundus images in accordance with the International Clinical Diabetic Retinopathy (ICDR) severity scale. In cases of grading discrepancy, a third senior retinal specialist (≥20 years’ experience) adjudicated the findings. Patients were classified as having RDR or non-RDR based on the worst eye. Data were entered into EDC electronic databases, with range and logic checks to minimize entry errors.

### 2.2 Study design

**Figure 1:**
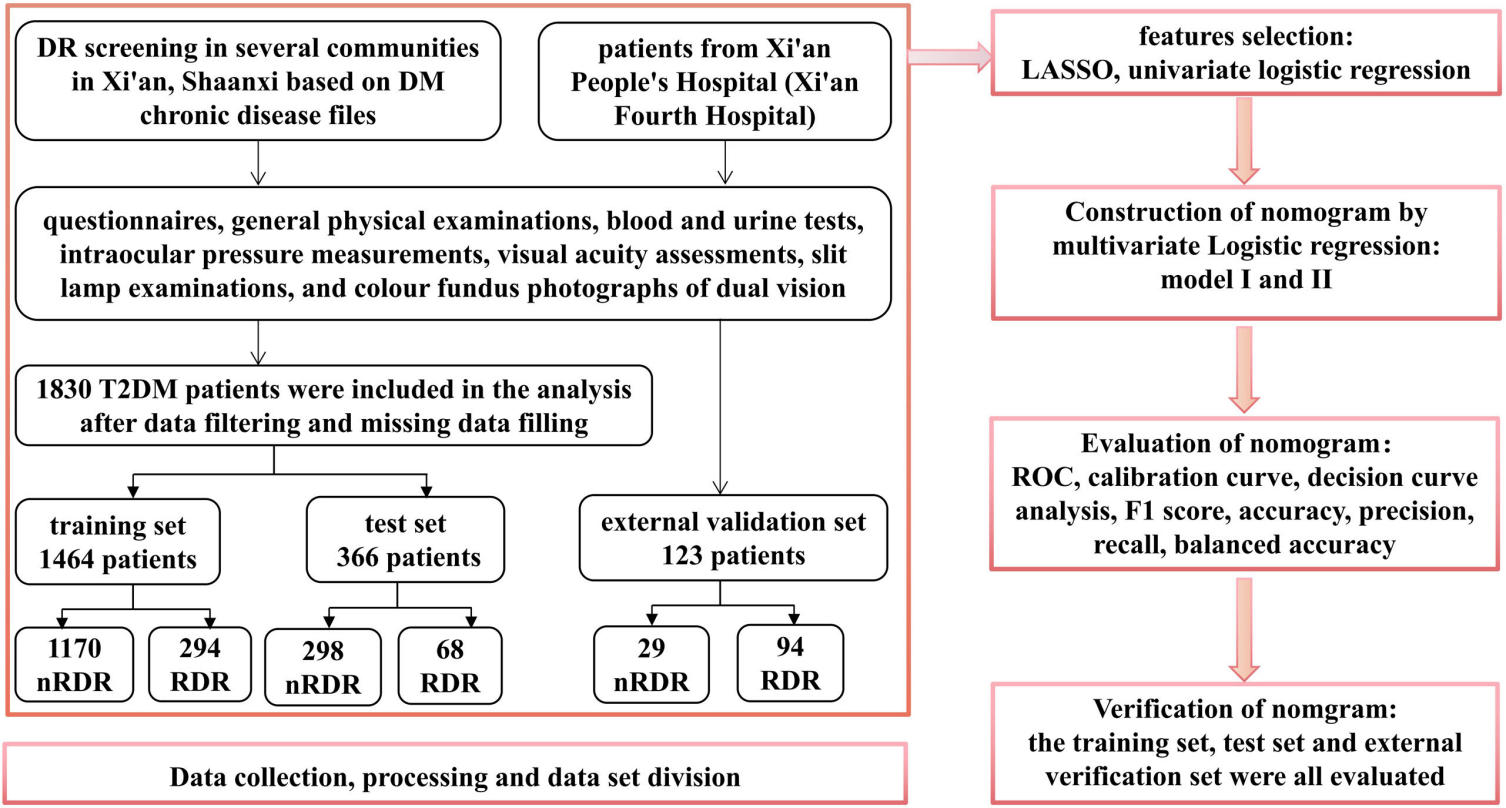
Diagrammatic representation of the study protocol.

This investigation analyzed 56 indicators from T2DM patients receiving care at 14 community health service centers in Xi’an, Shaanxi, China. Four variables exhibiting missing rates above 30% (postprandial blood glucose (PBG), hypertension, heart disease, and hyperlipidemia) were omitted from the analysis. For data with missing rates below 30%, imputation was performed using the random forest technique. The dataset from the community cohort (N = 1,830) was randomly split into a training set (80%, N = 1,464) for model development and an internal test set (20%, N = 366) for validation of performance. External validation was conducted using an additional 123 samples from Xi’an People’s Hospital (Xi’an Fourth Hospital), a tertiary hospital. This external test assesses the model’s generalizability to a different patient population and care setting. **Figure 1** provides a detailed illustration of the research methodology.

### 2.3 Statistical analysis

Statistical analyses were executed using R software (version 4.2.3, Vienna, Austria) and Python (version 3.7). Continuous variables were represented as mean ± standard deviation or median with interquartile range (IQR), while categorical variables were expressed as frequencies and percentages. Group comparisons for continuous variables were conducted using the t-test or Mann–Whitney U test as appropriate, whereas categorical variables were analyzed using the chi-square test. Prior to multivariable modeling, we systematically evaluated potential multicollinearity among predictors using both correlation matrices (visualized via heatmaps). Variables demonstrating high multicollinearity (Pearson correlation coefficient r > 0.9) were excluded from further analysis to avoid compromising model accuracy.

The univariate comparisons between the RDR group and non-RDR group within the training set, were first performed using appropriate tests (Student’s t-test or Mann-Whitney U for continuous variables, and chi-square for categorical variables) to identify factors significantly associated with RDR. All variables with *P* < 0.05 in univariate analysis were considered candidates for multivariable modeling. To further select predictors and avoid overfitting, the least absolute shrinkage and selection operator (LASSO) method were applied on the training data, using 10-fold cross-validation to determine the optimal regularization parameter (λ) (23). LASSO is a penalized regression technique that shrinks less important feature coefficients toward zero, effectively performing variable selection. We evaluated the path of coefficients and the cross-validated error to choose a parsimonious set of predictors (at the λ that minimizes the cross-validation error within one standard error of the minimum). By including all significant covariates in the multivariable model, we controlled for potential confounders; each predictor’s effect was thus estimated while adjusting for the others. Using the predictors retained by LASSO, we fitted a multivariable logistic regression model to the training set to estimate the probability of RDR. The regression coefficients were then used to construct a nomogram, a graphical prediction tool that assigns a point score to each predictor value and yields an individualized risk. The formula for the logistic model (logit function) was presented.

Model performance was evaluated using multiple metrics, including area under the receiver operating characteristic curve (AUROC), accuracy, precision, recall, F1 score, balanced accuracy, Youden index, calibration curves, and decision curve analysis (DCA). Discrimination ability was assessed via AUROC, with values exceeding 0.7 considered indicative of good performance (19). Calibration and clinical utility were evaluated using calibration curves and decision curve analysis, respectively (20, 24, 25). Model calibration, the agreement between predicted probabilities and observed outcomes, was assessed by calibration curves, where predictions were grouped into deciles and plotted against actual outcome proportions; a calibration curve close to the 45° line indicates good calibration. DCA examines the net benefit of using the model across a range of threshold probabilities at which a clinician would intervene. Net benefit is calculated by weighing true positives against false positives, relative to strategies of referring all patients vs. referring none. From the DCA, we identified the range of risk thresholds where the nomogram provides greater net benefit than “treat all” or “treat none” approaches. This range informs where the model is useful in practice. All statistical tests were two-tailed, with statistical significance set at *P* < 0.05.

## 3 Results

### 3.1 Baseline characteristics

A total of 1,830 patients with type 2 diabetes were included in the internal cohort (training + test), of whom 362 (19.78%) diagnosed as RDR. An 80:20 ratio was employed to randomly allocate samples into training and test sets. The training set encompassed 1,464 patients, including 294 (20.08%) RDR cases, while the test set comprised 366 individuals, of which 68 (18.58%) were RDR cases. The external validation cohort (N = 123) consisted of T2DM outpatients, with 23 RDR cases (18.70%) (**Supplementary Table S1**).

**Figure 2:**
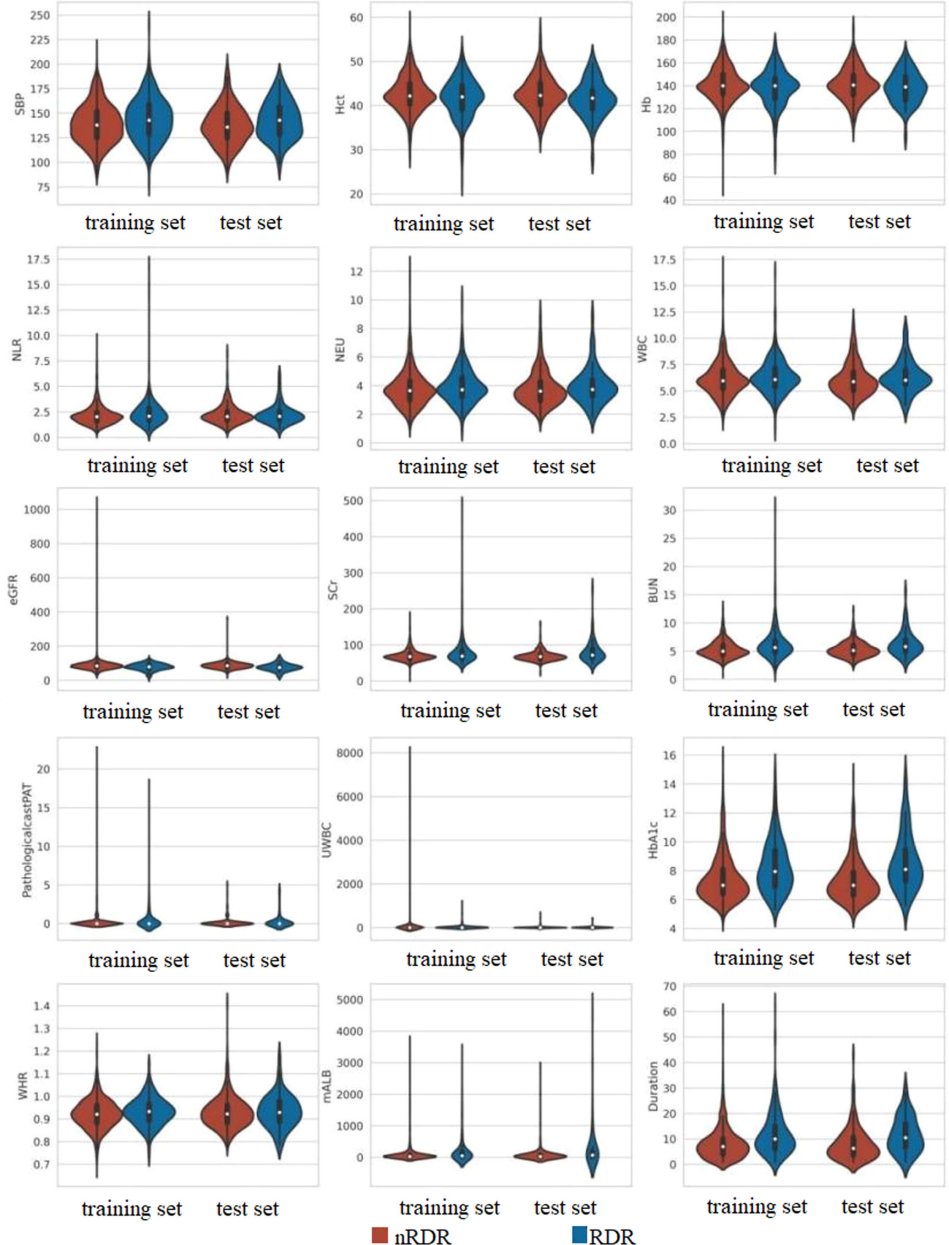
Comparative distribution analysis of continuous variables between training and test datasets.

**Figure 3:**
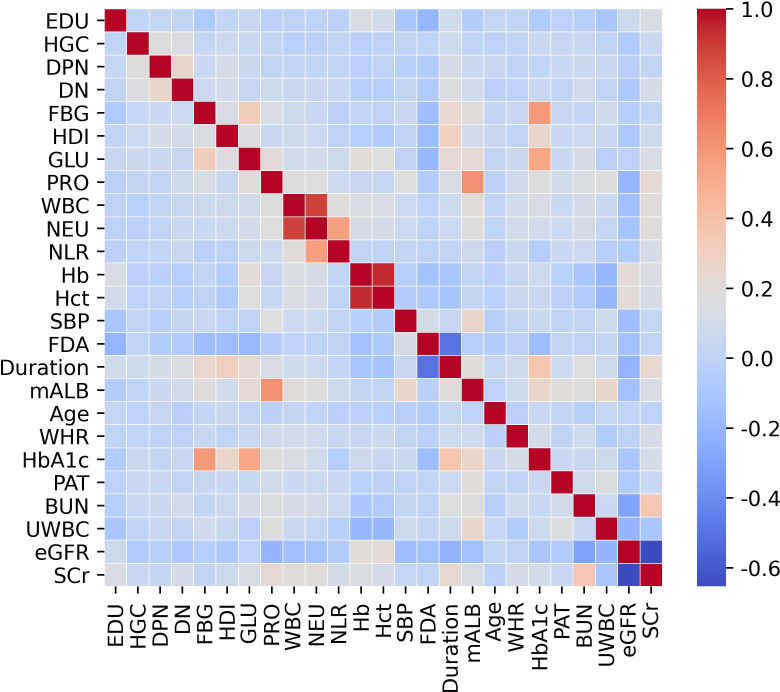
Correlation matrix of variables within the training cohort.

We initially examined 52 candidate variables for association with RDR. Comparison of baseline variables between RDR and non-RDR groups in the combined internal cohort revealed significant differences in multiple demographic and clinical factors. Patients with RDR tended to have lower educational attainment, a higher prevalence of glycosuria, and more frequent use of hypoglycemic drugs or insulin (HDI) compared to non-RDR patients. Poor fasting blood glucose control was more common in the RDR group, as were diabetic nephropathy (DN) and peripheral neuropathy (DPN) diagnoses. A history of hypoglycemic coma (HGC) was also more prevalent among RDR cases (*P* < 0.05). In terms of continuous variables, the RDR group had significantly higher median systolic blood pressure (SBP) and a higher inflammatory cell count (neutrophils and white blood cell count), reflected in a mildly elevated neutrophil-lymphocyte ratio (NLR). Markers of renal function were worse in RDR patients: median serum creatinine (SCr) and blood urea nitrogen (BUN) were higher, while estimated glomerular filtration rate (eGFR) was lower in RDR vs non-RDR. Likewise, urine microalbumin (mALB) was markedly elevated in the RDR group (median 52.9 vs 27.2 mg/L) and proteinuria (PRO) was more frequent (37.3% vs 23.6%; *P* < 0.001) in RDR patients. Glycemic indices differed as expected: RDR patients had higher HbA1c (median 8.0% vs 7.0%) and a greater waist-to-hip ratio (WHR) than non-RDR (both *P* < 0.05). Notably, RDR patients had longer diabetes duration (median 10.0 vs 6.5 years) and were diagnosed with diabetes at a younger age on average (first diagnosis age ~56.0 vs 60.4 years; *P* < 0.001). These results underscore that the RDR group had more adverse profiles in glycemic control, blood pressure, renal function, and diabetes chronicity (**Table 1**). Violin plots illustrate continuous variables between groups (**Figure 2**). A correlation analysis found that hematocrit and hemoglobin were highly collinear (r > 0.9) (**Figure 3**), so hemoglobin was excluded from further modeling to avoid multicollinearity.

**Table 1:** Demographic and clinical characteristics of the training and test sets.

Variables		Non-RDR (n=1468)	RDR (n=362)	Statistics	P value
Educational level, n(%)	no formal school education	136(9.26)	29(8.01)	10.64	0.014
	Junior high school	843(57.43)	237(65.47)		
	High school, technical secondary school, technical school	332(22.62)	74(20.44)		
	College graduate, undergraduate graduate, graduate student or above	157(10.69)	22(6.08)		
Income, n(%)	< 1000 RMB	311(21.19)	79(21.82)	4.50	0.212
	1000-2999 RMB	631(42.98)	174(48.07)		
	3000-5999 RMB	453(30.86)	94(25.97)		
	> 6000 RMB	73(4.97)	15(4.14)		
Smoke, n(%)	no	1243(84.67)	307(84.81)	0.00	0.950
	yes	225(15.33)	55(15.19)		
Drink, n(%)	no	1291(87.94)	326(90.06)	1.26	0.262
	yes	177(12.06)	36(9.94)		
Activity, n(%)	no	1327(90.40)	336(92.82)	2.06	0.152
	yes	141(9.60)	26(7.18)		
Gender, n(%)	no	870(59.26)	215(59.39)	0.00	0.965
	yes	598(40.74)	147(40.61)		
NIT, n(%)	no	1394(94.96)	341(94.20)	0.34	0.559
	yes	74(5.04)	21(5.80)		
URO, n(%)	no	1438(97.96)	359(99.17)	2.42	0.120
	yes	30(2.04)	3(0.83)		
PRO, n(%)	no	1121(76.36)	227(62.71)	27.91	<0.001
	yes	347(23.64)	135(37.29)		
BLO, n(%)	no	1155(78.68)	287(79.28)	0.06	0.801
	yes	313(21.32)	75(20.72)		
KET, n(%)	no	1426(97.14)	350(96.69)	0.21	0.648
	yes	42(2.86)	12(3.31)		
GLU, n(%)	no	1114(75.89)	187(51.66)	82.95	<0.001
	yes	354(24.11)	175(48.34)		
HDI (Hypoglycemic drugs or insulin), n(%)	no	305(20.78)	39(10.77)	19.04	<0.001
	yes	1163(79.22)	323(89.23)		
FBG control, n(%)	<6.1mmol/L	138(9.40)	23(6.35)	68.14	<0.001
	6.2-7.2mmol/L	623(42.44)	111(30.66)		
	7.3-8.8mmol/L	483(32.90)	105(29.01)		
	>8.8mmol/L	224(15.26)	123(33.98)		
DN, n(%)	no	1392(94.82)	324(89.50)	14.07	<0.001
	yes	76(5.18)	38(10.50)		
DPN, n(%)	no	1294(88.15)	301(83.15)	6.48	0.011
	yes	174(11.85)	61(16.85)		
DCD, n(%)	no	1248(85.01)	302(83.43)	0.57	0.452
	yes	220(14.99)	60(16.57)		
DK, n(%)	no	1459(99.39)	359(99.17)	0.21	0.649
	yes	9(0.61)	3(0.83)		
HGC, n(%)	no	1426(97.14)	342(94.48)	6.30	0.012
	yes	42(2.86)	20(5.52)		

HDI, use of hypoglycemic drugs or insulin; FBG control, fasting blood glucose control; DN, diabetic nephropathy; DPN, diabetic peripheral neuropathy; DCD, diabetic cardiovascular disease; DK, diabetic ketoacidosis; HGC, hypoglycemic coma; WHR, waistline to hipline ratio; URBC, the number of red blood cells in urine; Uabnormal RBC, the number of the abnormal red blood cells in urine; UWBC, the number of white blood cells in urine; Hyahyaline cast, PAT, pathological cast; NIT, nitrite; URO, urobilinogen; PRO, protein in urine; BLO, urine occult blood; KET, ketone; GLU, glucose in urine; TC, total cholesterol; TG, triglyceride; HDL-C, high-density lipoprotein cholesterol; BUN, urea nitrogen; UA, uric acid; SCr, serum creatinine; eGFR, estimated glomerular filtration rate; BMI, body mass index; WBC, white blood cell; NEU, neutrophil; LYM, lymphocyte; NLR, neutrophil to lymphocyte ratio; RBC, red blood cell; Hb, haemoglobin; Hct, hematocrit; SBP, systolic pressure; DBP, diastolic pressure; FDA, age at first diagnosis of diabetes; mALB, microalbuminuria.

### 3.2 Risk factors screening

**Figure 4:**
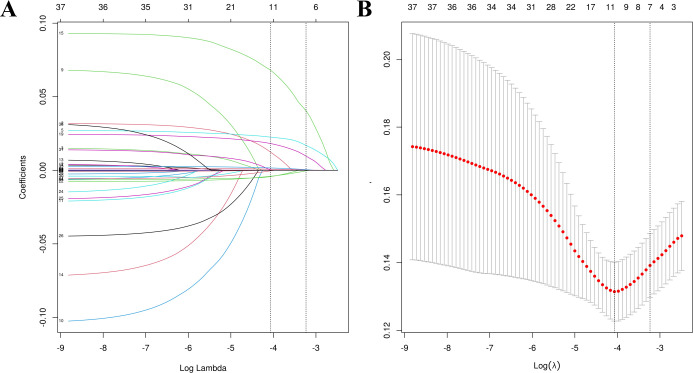
Feature selection utilizing LASSO regression. **(A)** Optimal lambda parameter identifying four variables with non-zero coefficients. **(B)** Visualization of partial likelihood deviance against log(lambda), with vertical dashed lines indicating the 1-SE criterion.

The training set patients were classified into RDR and non-RDR groups. Univariate analysis in the training set confirmed that numerous factors were significantly associated with RDR status (*P* < 0.05), including higher SBP, BUN, NEU, NLR, SCr, mALB, HbA1c, and presence of glycosuria, as well as longer diabetes duration and histories of DN, DPN, poor fasting glycemic control, hypoglycemic coma, and proteinuria (**Table 2**). These variables were then subjected to LASSO regression analysis (**Figure 4**). Ten-fold cross-validation optimized the regularization parameter (λ). **Figure 4** demonstrates that increasing λ intensifies variable shrinkage penalties, reducing the number of selected variables. **Figure 4A** illustrates coefficient changes for each variable as λ increases, along with predictor numbers. **Figure 4B** displays λ logarithmic values (horizontal axis), error values (vertical axis), and selected predictor numbers for each λ value. Dashed lines indicate optimal λ values (λ.min and λ.1se). Two Gaussian LASSO regression models were developed based on different λ criteria. Model I, based on λ.min (λ = 0.002), incorporated ten non-zero coefficient predictors: eGFR, age, SCr, BUN, GLU, HbA1c, mALB, duration, SBP, and NLR (**Supplementary Table S2**). Model II, using λ.1se (λ = 0.03), included seven non-zero coefficient predictors: SCr, BUN, GLU, HbA1c, mALB, duration, and SBP (**Supplementary Table S3**). Each of these predictors showed a positive association with RDR risk in multivariate analysis in Model II.

**Table 2:** Univariate analysis of variables associated with RDR.

Indicators	N	OR	95%CI	P-value
eGFR	1830	0.978	[0.972,0.985]	0.000
BUN	1830	1.299	[1.217,1.386]	0.000
NEU	1830	1.024	[0.974,1.077]	0.346
NLR	1830	1.123	[1.028,1.227]	0.010
SBP	1830	1.013	[1.007,1.019]	0.000
mALB	1830	1.001	[1.001,1.002]	0.000
Duration	1830	1.072	[1.055,1.088]	0.000
FDA	1830	0.998	[0.995,1.001]	0.287
Age	1830	1.014	[0.999,1.029]	0.072
SCr	1830	1.023	[1.017,1.029]	0.000
HbA1c	1830	1.350	[1.266,1.44]	0.000
PAT	1830	1.138	[1.027,1.263]	0.014
UWBC	1830	1.000	[0.999,1.0]	0.733
EDU				
0 no formal school education	165			
1 Junior high school	1092	1.307	[0.854,2.001]	0.218
2 High school, technical secondary school, technical school	394	1.066	[0.663,1.714]	0.790
3 College graduate, undergraduate graduate, graduate student or above	179	0.657	[0.361,1.197]	0.170
GLU				
0 no	1301			
1 yes	529	2.945	[2.320,3.737]	0.000
HGC				
0 no	1768			
1 yes	62	1.986	[1.151,3.426]	0.014
DPN				
0 no	1599			
1 yes	231	1.547	[1.126,2.127]	0.007
DN				
0 no	1717			
1 yes	113	2.374	[1.585,3.556]	0.000
FBG				
0.0 <6.1mmol/L	161			
1.0 6.2-7.2mmol/L	727	1.058	[0.651,1.721]	0.819
2.0 7.3-8.8mmol/L	599	1.305	[0.801,2.127]	0.286
3.0 >8.8mmol/L	343	3.355	[2.048,5.495]	0.000
HDI				
0 no	338			
1 yes	1492	2.266	[1.576,3.257]	0.000
PRO				
0 no	1348			
1 yes	482	1.892	[1.481,2.416]	0.000

HDI, use of hypoglycemic drugs or insulin; FBG control, fasting blood glucose control; DN, diabetic nephropathy; DPN, diabetic peripheral neuropathy; DCD, diabetic cardiovascular disease; DK, diabetic ketoacidosis; HGC, hypoglycemic coma; WHR, waistline to hipline ratio; URBC, the number of red blood cells in urine; Uabnormal RBC, the number of the abnormal red blood cells in urine; UWBC, the number of white blood cells in urine; Hyahyaline cast, PAT, pathological cast; NIT, nitrite; URO, urobilinogen; PRO, protein in urine; BLO, urine occult blood; KET, ketone; GLU, glucose in urine; TC, total cholesterol; TG, triglyceride; HDL-C, high-density lipoprotein cholesterol; BUN, urea nitrogen; UA, uric acid; SCr, serum creatinine; eGFR, estimated glomerular filtration rate; BMI, body mass index; WBC, white blood cell; NEU, neutrophil; LYM, lymphocyte; NLR, neutrophil to lymphocyte ratio; RBC, red blood cell; Hb, haemoglobin; Hct, hematocrit; SBP, systolic pressure; DBP, diastolic pressure; FDA, age at first diagnosis of diabetes; mALB, microalbuminuria.

### 3.3 Construction and evaluation of nomogram

Using the coefficients, A nomogram was constructed to facilitate the visualization of the diagnostic model and predict the probability of RDR in an accessible manner. The performance of nomograms were evaluated from the following aspects.

**Figure 5:**
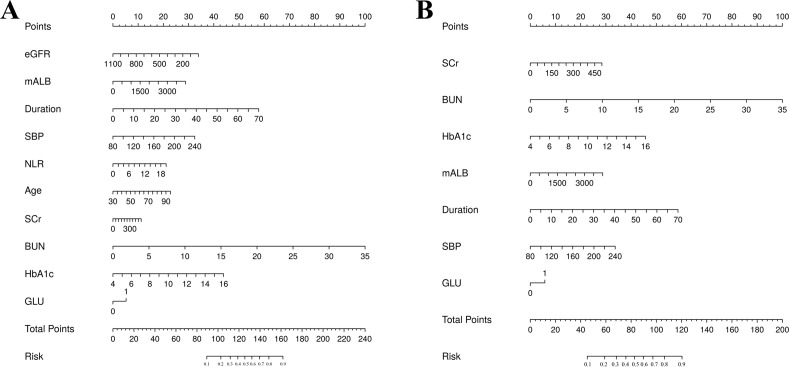
Risk nomogram Model I **(A)** and Model II **(B)** for identifying RDR in T2DM patients.

**Figure 6:**
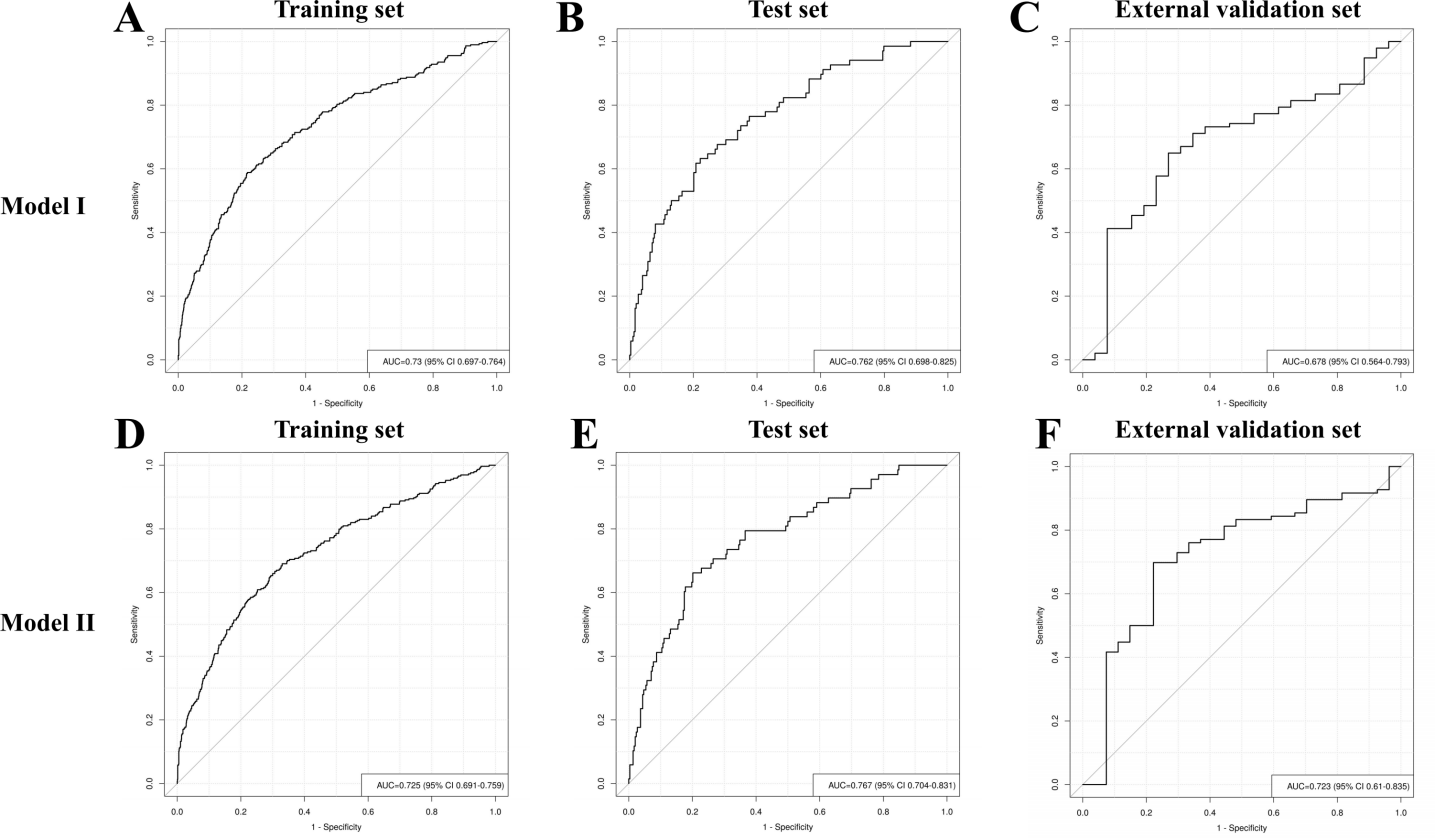
Receiver operating characteristic curve analysis of RDR risk prediction Model I and Model II in the training set **(A, D)**, test set **(B, E)** and external validation set **(C, F)**.

Discrimination: Model I was developed based on the aforementioned 10 variables (**Figure 5A**). The AUCs were 0.730 (95% CI: 0.697–0.764), 0.762 (95% CI: 0.698–0.825), and 0.678 (95% CI: 0.564–0.793) for the training, test, and external validation sets, respectively (**Figures 6A–C**). Model II was constructed utilizing the seven variables mentioned above (**Figure 5B**). The AUCs were 0.730 (95% CI: 0.691–0.759) for the training set, 0.767 (95% CI: 0.704–0.831) for the test set, and 0.723 (95% CI: 0.610–0.835) for the external validation set (**Figures 6A–C**), indicating consistent moderate accuracy. The results indicate that both models achieve moderate discriminative power. For context, Model II’s AUCs were comparable to Model I in the internal data (~0.730 in training and ~0.760 in test) but notably higher on external validation (0.723 vs 0.678 for Model I), suggesting Model II generalized better to new patients.

Classification metrics: The detailed performance metrics for the two models across the three datasets are summarized in **Table 3**. Each model was evaluated in terms of accuracy, precision, recall, F1 score, balanced accuracy, Youden index, and cutoff, as presented in **Table 4**. Using the optimal probability cutoff derived from the training set (Youden’s index optimized at 0.186 for Model II), we evaluated the sensitivity, specificity, and predictive values. In the training set, Model II correctly identified 66.7% of RDR cases (sensitivity 66.7%) while correctly excluding 82.4% of non-RDR cases (specificity 82.4%). The overall accuracy in the training data was 81.6%, and the balanced accuracy (average of sensitivity and specificity) was 74.6%. Owing to the low prevalence of RDR, the positive predictive value (PPV) was modest (17.0%), whereas the negative predictive value (NPV) was very high (97.8%), indicating that a patient predicted as low-risk by the model is very likely to truly be non-RDR. The Youden index for the training model was 0.382, confirming a good trade-off between sensitivity and specificity at the chosen cutoff. When applied to the internal test set (N = 366, 18.6% RDR prevalence), Model II maintained similar performance. The AUC of 0.767 was essentially identical to training, and at the same probability threshold (0.186), sensitivity was ~56% and specificity ~84%. The PPV was ~20.6% and NPV ~96.3% in test set, consistent with expectations given the slightly lower RDR prevalence in the test sample. In the external validation set (N = 123, 18.7% RDR), the nomogram’s discrimination remained good (AUC 0.723). Applying the fixed 0.186 cutoff to this cohort yielded a high sensitivity of 92.8% with specificity of 26.3%. This indicates that the model identified nearly all actual RDR cases in the external sample (only ~7% false negatives), at the expense of more false positives among non-RDR patients. The PPV in external validation was 27.1%, while the NPV was 92.6%. The lower specificity and PPV in the external set reflect that the nomogram, calibrated on the internal cohort, tended to over-predict RDR for the new population. Nonetheless, the high NPV and sensitivity in external validation underscore that Model II is effective as a screening tool -it rarely misses true RDR cases, even if it flags some false positives. Given that missing an RDR case has greater clinical consequence (potentially untreated retinopathy) than over-referral, this operating profile is acceptable for a referral triage model. Overall, the model’s discrimination and classification metrics indicate robust performance, with Model II showing slightly improved generalizability relative to the more complex Model I.

**Table 3:** Detailed performance metrics for the three datasets in Model I and Model II.

	Model I			Model II		
	Training set	Test set	External validation set	Training set	Test set	External validation set
AUC	0.730	0.762	0.678	0.730	0.767	0.723
Sensitivity	0.675	0.571	0.894	0.667	0.56	0.928
Specificity	0.827	0.846	0.231	0.824	0.842	0.263
PPV	0.184	0.235	0.175	0.17	0.206	0.271
NPV	0.978	0.544	0.923	0.978	0.963	0.926

AUC, area under the curve; PPV, positive predictive value; NPV, negative predictive value.

**Table 4:** Different indicators for evaluating the effectiveness of RDR risk prediction models.

Model	Accuracy	Precision	Recall	F1-score	Balanced accuracy	Youden index	Cut-off
I	0.818	0.184	0.675	0.289	0.751	0.372	0.232
II	0.816	0.170	0.667	0.271	0.746	0.382	0.186

Stepwise forward regression analysis identified ten independent predictors for Model I and seven independent predictors for Model II. **Supplementary Tables S2** and **S3** present the coefficients of the covariates in both models. In the multivariate analysis, two logistic regression models (Model I and Model II) were constructed for RDR prediction using different predictor combinations. An equation was derived to estimate the probability (*P*) of RDR occurrence based on the coefficients of the significant predictors as follows:

Model I:


  logit(P)=−0.872+(−0.002)×eGFR+0×mALB+0.050×Duration+0.012×SBP+0.064×NLR+0.021×Age+0.001×SCr+0.173×BUN+0.222×HbA1c+0.317×GLU

Model II:


 logit(P)=−0.672+0×mALB+0.049×Duration+0.012×SBP+0.003×SCr+0.167×BUN+0.222×HbA1c+0.338×GLU

where logit(*P*) is the linear predictive value (log-odds of RDR). The *P* (probability) of RDR for a given patient is then obtained as:


$$P=\frac{e^{\text{logit}\left(P\right)}}{1+e^{\text{logit}\left(P\right)}}$$

e is Euler’s number, approximately 2.71828.

**Figure 7:**
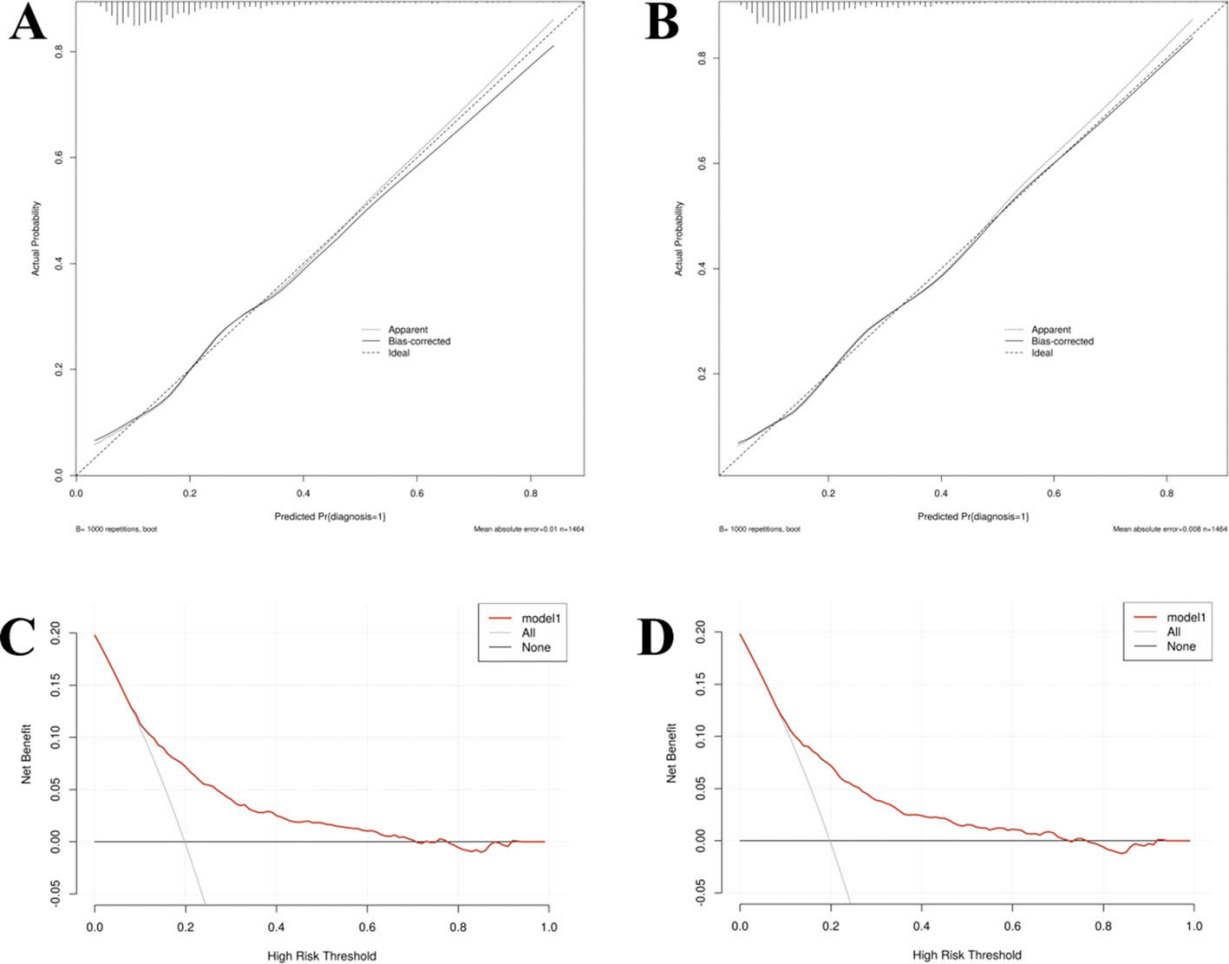
The calibration curve **(A, C)** and decision curves analysis **(B, D)** of Model I and Model II in the training set.

Calibration: Calibration curves were employed to evaluate the accuracy of the prediction models, demonstrating that the predicted probabilities closely corresponded to the actual probabilities in Models I and II (**Figures 7A, B**).

Decision curve analysis: Decision curve analysis (DCA) indicated that the threshold probability for identifying referable diabetic retinopathy (RDR) ranged from 8% to 70% for Model I and 8% to 72% for Model II (**Figures 7C, D**). Within these respective ranges, the application of both nomogram models provides greater clinical net benefit compared with the alternative strategies of universally referring all patients (“treat-all”) or none (“treat-none”). In clinical practice, the threshold probability reflects the predicted risk at which a clinician would typically consider intervening (i.e., referring the patient to an ophthalmologist for detailed examination). The wide threshold probability range for Model II (8%-72%) indicates its applicability across diverse clinical scenarios.

For instance, if a clinician chooses a threshold of 20% predicted risk to trigger referral (a moderately conservative choice), the decision curve indicates the nomogram will improve patient outcomes by identifying high-risk individuals for timely specialist eye evaluation while avoiding unnecessary referrals in low-risk patients. In practical terms, applying Model II means that patients with a predicted RDR risk above the clinician’s chosen cutoff would be referred for prompt retinal examination (potentially preventing vision loss through early treatment), whereas those below the threshold could be monitored routinely in primary care. Thus, the nomogram provides clinical net benefit over a broad range of decision thresholds, supporting its value as a risk stratification tool for guiding referral decisions. Ultimately, the robust performance and fewer input variables required for Model II support its selection as the finalized nomogram. Its demonstrated utility across a wide range of clinical decision-making scenarios emphasizes its practical value for personalized diabetic retinopathy management, facilitating precise and efficient care delivery.

Risk stratification across DR severity: To further assess the model’s behavior, we analyzed the distribution of nomogram scores and predicted risk across different DR severity levels. Among patients who had RDR, higher risk scores were correlated with more advanced DR stages. In the internal cohort’s RDR cases (moderate NPDR, severe NPDR, or PDR), the nomogram score was significantly higher in severe NPDR cases compared to moderate NPDR (median points 68.97 vs 62.82, *P* = 0.006). Correspondingly, the median predicted probability of RDR in moderate NPDR was 23.5% (IQR 13.8–37.2%), rising to 30.5% (IQR 17.3–51.3%) in severe NPDR cases. This trend was even more evident in the external validation set’s RDR patients. In the external cohort, the nomogram’s median predicted risk was only 11.8% for those with moderate NPDR, compared to 22.0% in severe NPDR and 48.2% in PDR (overall *P* < 0.001 across stages) (**Supplementary Tables S4-S8**). Thus, the model assigned substantially higher risk probabilities to individuals with sight-threatening DR (severe NPDR or PDR) than to those with milder RDR. These findings indicate that Model II not only differentiates patients with any referable retinopathy from those without, but it also stratifies risk within the RDR spectrum, aligning with disease severity. In clinical terms, a higher nomogram score signals not just the presence of RDR but likely a more advanced stage of retinopathy, which could help prioritize patients by urgency. The consistency of this pattern in both internal and external sets supports the model’s validity and its potential utility in identifying patients at highest risk of advanced DR progression.

### 3.4 Clinical utility of the nomogram

**Figure 8:**
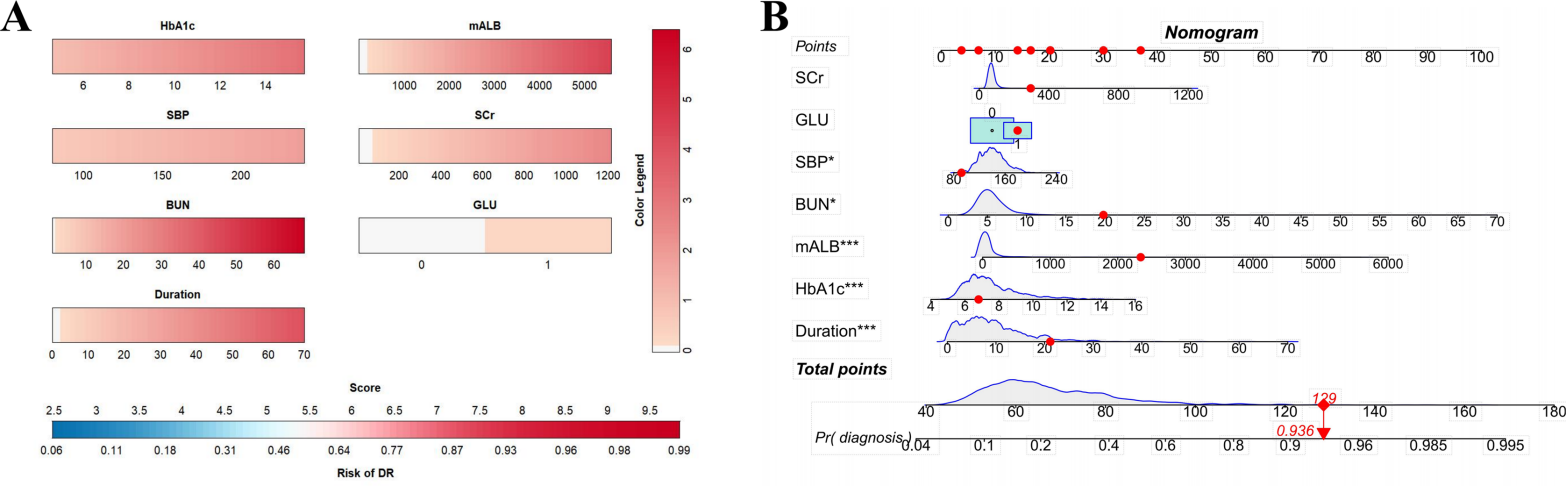
**(A)** Referable diabetic retinopathy (RDR) risk nomogram. Red indicates that the variable is a risk factor for RDR, and blue indicates that the variable is a protective factor for RDR. The darker the color, the greater the influence of the variable on RDR. **(B)** Dynamic nomogram. A dynamic nomogram based on Model II was created to predict the risk of developing RDR in T2DM patients. According to the patient’s SCr (100 μmol/L), HbA1c (8.2%), SBP (138 mmHg), GLU(1, positive), BUN (10.7 mmol/L), mALB (2339.27 ug/mg), duration (24 years), the total points was 129, and the predicted probability of RDR was 0.936. Therefore, the patient had an 93.6% chance of developing RDR.

A dynamic nomogram was constructed using the coefficients from Model II (**Figure 8A**) to evaluate and illustrate the risk of developing RDR in T2DM patients (**Figure 8B**). Numerical values were assigned to each risk factor. To determine the points for each factor, a perpendicular line was drawn from its corresponding value to the “Points” axis. The points from all factors were subsequently summed to calculate the Total Score, and a vertical line was drawn to the “Total Score” row to assess the probability of RDR occurrence. An exemplar patient from the external dataset is highlighted in red. The varying dimensions of the rectangles demonstrate the differences in the relative proportion of patients across each subgroup (**Figure 8B**).

## 4 Discussion

In this study, we developed and validated a parsimonious nomogram (Model II) for predicting referable diabetic retinopathy in patients with T2DM. The model integrates seven readily available clinical and laboratory variables and demonstrated good discriminative performance. Importantly, we externally validated the model on an independent cohort, confirming its generalizability. The nomogram provides individual risk estimates for moderate-or-worse DR in a form that is easily interpretable by clinicians. Using a risk score tool, frontline providers can stratify diabetic patients by RDR risk and guide timely ophthalmology referrals. This approach addresses the pressing need for scalable, cost-effective DR screening strategies, as traditional fundus examination programs often fail to cover all at-risk patients. To our knowledge, this is one of the first RDR prediction models in a Chinese population that combines community health data with hospital data and undergoes external validation, supporting its broader applicability.

### 4.1 Innovation and comparison to prior models

Our study offers a significant advancement in diabetic retinopathy (DR) risk prediction by developing a clinically applicable nomogram that achieves robust discriminative performance (AUC ≈ 0.73) using only routinely collected clinical parameters, including HbA1c, systolic blood pressure, renal function markers, and urine glucose. Unlike prior models that often rely on specialized, costly, or technologically demanding indicators, our model is optimized for scalability and accessibility in real-world settings, particularly in primary care. A comparative analysis with existing models (**Table 5**) highlights this distinction. For instance, Ke et al. (19) utilized 2-hour C-peptide levels and nerve conduction assessments, achieving an AUC of 0.75 but at the cost of requiring non-routine testing (19). Wang et al. (29) reported higher predictive accuracy (AUC > 0.80) using insulin resistance indices and vitamin D levels, yet these biomarkers are not standard in most clinical workflows (20). Conversely, community-based models by Pan et al. (21) and Mo et al. (28) employed readily available variables (AUC ~0.70) but lacked external validation, limiting their generalizability (15, 21).

**Table 5:** Comparison of predictive models for DR in T2DM patients.

Study (Year)	Train and Test set	External Validation	Outcome	Predictors (n)	Performance
Pan et al., 2023 (21)	Community, Shanghai, China (N=2385)	No	DR	6 predictors: indglycosylated hemoglobin A1c, disease course, postprandial blood glucose, age, systolic blood pressure, and albumin/urine creatinine ratio	AUC (0.703), accuracy (0.796), precision (0.571), recall (0.035), F1 score (0.066), Hosmer-Lemeshow test (0.887), NRI (0.004), and balanced accuracy (0.514).
Tao et al., 2023 (26)	Hospital, Shanghai, China (N=788)	No	DR	Continuous glucose monitoring (CGM) data	AUC: 0.86 (training); 0.85 (test)
Ke et al., 2023 (19)	Hospital, Beijing, China (N=440)	Yes (external validation, Hospital in Beijing, China N=120)	VTDR	3 predictors: 2-h C-peptide, UACR, sural nerve conduction impaired (SNCI)	AUC: 0.76 (training), 0.73 (test), 0.75 (external validation)
Yang et al., 2023 (16)	Hospital, Chongqing, China (N=4159)	Yes (external validation, Hospital in Chengdu, China, N=430)	DR	3 predictors: the duration of diabetes, history of hypertension, and cardiovascular disease	AUC: 0.722 (training), 0.715 (internal test), and 0.703 (external test); DCA threshold probability:17-55%
Wang et al., 2023 (20)	Hospital, Zunyi, China (N=213)	No	DR	8 predictors: disease duration, BMI, fasting blood glucose, HbA1c, homeostatic model assessment-insulin resistance (HOMA-IR), TG, total cholesterol (TC), and vitamin D (VitD)-T3	C-index: 0.848 (95% CI: 0.798-0.898) (training); 0.816 (interval validation)
Liu et al., 2023 (27)	Hospitals, Gansu, China (N=520)	No	DR	8 predictors: age, DPN, HbA1C, HDL-C, NLR, TG, BUN, and disease duration	AUC: 0.773 (training) and 0.735 (test); DCA threshold probability: 11%-95% (training) and 17%-93% (test)
Yang et al., 2022 (15)	Hospital, Shijiazhuang, China (N=5900)	No	DR	8 predictors:duration of diabetes, diabetic neuropathy, diabetic kidney disease, diabetic foot, hyperlipidemia, hypoglycemic drugs, glycated albumin, Lactate dehydrogenase	AUC: 0.820 (training); 0.842 (test); 2% and 75% (training) ; DCA threshold probability: 2%-88%(validationt)
Li et al., 2022 (17)	Hospitals, Xinjiang, China (N=13980)	No	DR	7 predictors: DPN, age, neutrophilic granulocyte (NE), HDL-C, HbA1C, duration of T2DM, and glycosylated serum protein (GSP)	AUC: 0.882 (95% CI, 0.875-0.888) (development); 0.870 (95% CI, 0.856-0.881) (validation)
Mo et al., 2020 (28)	Community, Shanghai, China (N=4170)	No	DR	7 predictors: age, course of disease, postprandial blood glucose (PBG), HbA1c, uric creatinine (UCR), urinary microalbumin (UMA), and SBP	AUC: 0.700 (training); 0.715 (validation)
Present study (2025)	Community, Xi'an, China (N=1830)	Yes (external cohort from hospital in Xi'an, China) (N=123)	RDR	7 predictors: duration, HbA1c, urine glucose, SCr, BUN, mALB, SBP	AUC: 0.730 (95% CI: 0.691–0.759) (training); 0.767 (95% CI: 0.704–0.831) (test), and 0.723 (95% CI: 0.610–0.835) (external validation); DCA threshold probability: 8%-72%

BMI: body mass index; FPG: fasting blood glucose; PBG: postprandial blood glucose; TGs: total triglycerides; SBP: systolic blood pressure; VTDR: vision-threatening diabetic retinopathy; DPN: Diabetic Peripheral Neuropathy; BUN: Blood Urea Nitrogen; HDL-C: high-density lipoprotein; NLR: Neutrophil-to-Lymphocyte Ratio;

In contrast, our nomogram is built upon a large, community-derived dataset and validated externally in an independent tertiary hospital cohort, ensuring consistent performance across diverse healthcare settings and patient populations. This dual-source design, encompassing both general T2DM populations with early or absent retinopathy and ophthalmic outpatients with more advanced disease, captures a broad spectrum of DR severity (**Supplementary Tables S4–S8**), enhancing model robustness and applicability.

A key strength of our model lies in its explicit focus on referable DR (RDR), a clinically actionable endpoint that directly informs ophthalmologic referral decisions. While many previous models predict general DR risk without regard to severity, our model offers immediate utility in clinical triage by distinguishing those at risk for vision-threatening stages. Furthermore, by employing LASSO regression for variable selection, we identified a parsimonious set of seven predictors that preserved model performance while enhancing interpretability and implementation feasibility.

The integration of community chronic disease registry data with hospital-based clinical records further underscores the model’s translational relevance and real-world alignment. This methodological approach bridges care levels, yielding a universal risk tool that can support both proactive identification of high-risk individuals in primary care and risk stratification of referred patients in ophthalmology settings.

In summary, Model II strikes a clinically meaningful balance between predictive accuracy, interpretability, and generalizability. Its exclusive reliance on universally obtainable variables, rigorous external validation, and focus on an actionable outcome distinguish it from existing models. This work contributes a practical, scalable framework for risk-based DR screening—particularly suited for integration into population health strategies and electronic medical record systems in resource-limited settings.

### 4.2 Pathophysiological and clinical implications

The composition of the final model carries implications for both understanding DR and managing it. Glycemic control and hypertension are well-established modifiable risk factors for DR. Elevated HbA1c and prolonged diabetes duration are associated with increased DR risk, reflecting chronic hyperglycemia’s detrimental effects on retinal microvasculature (23). Our findings echo landmark trials (DCCT, UKPDS) which showed that tighter glycemic control markedly reduces DR incidence and progression. For instance, every 1% reduction in HbA1c can lower the risk of DR complications by roughly 30%-35%% (27, 30). Notably, our model includes glycosuria as an independent predictor, suggesting that acute hyperglycemic episodes, indicated by urine glucose positivity, may contribute to retinal damage beyond chronic glycemic burden. This aligns with evidence that glycemic variability can induce oxidative stress and inflammatory responses in retinal tissues (31). Thus, the presence of glycosuria in a patient could identify those experiencing unrecognized hyperglycemic excursions, further elevating DR risk despite similar HbA1c levels. These findings align with the current clinical guidelines advocating tight blood glucose regulation as a cornerstone of DR risk reduction and overall diabetes management (32).

Renal function markers emerged as significant predictors in our nomogram, underscoring the interconnected nature of diabetic microvascular complications (33). In our study, elevated SCr, BUN, and mALB, were associated with higher RDR risk. These findings are consistent with studies that microalbuminuria is a marker for the presence and severity of DR (29, 34, 35). These findings support the concept that nephropathy and retinopathy share common pathogenic mechanisms, such as endothelial dysfunction and chronic inflammation (34, 36). Clinically, this underscores the value of monitoring renal status in diabetic patients not just to manage nephropathy, but also as a window into DR risk (37).

Hypertension, particularly elevated SBP, contributes to retinal capillary damage and ischemia, is a known exacerbating factor for DR. Our nomogram indicate increase in SBP was associated with higher RDR risk (38). Blood pressure control, particularly maintaining SBP in recommended ranges, has been shown to slow DR progression and is advocated by current guidelines. The epidemiologic data indicate that a 10 mmHg SBP reduction yields about a 13% decrease in DR risk. There is also evidence that fluctuations in blood pressure may impact DR progression. Effective blood pressure management is crucial in mitigating DR progression.

The prominence of glycemic control and blood pressure confirms that aggressive management of these modifiable factors remains central to preventing DR. Our findings reinforce existing guidelines that advocate tight control of HbA1c and blood pressure to reduce microvascular complications. Additionally, the inclusion of renal indicators (SCr, BUN, mALB) in the risk score highlights that clinicians should view abnormal kidney findings as a red flag for potential retinopathy. In practice, a diabetic patient with rising creatinine or significant albuminuria should prompt heightened vigilance for DR and perhaps expedited retinal screening. This interdependence of diabetic complications suggests a multidisciplinary care approach: close collaboration between endocrinologists, nephrologists, and ophthalmologists can facilitate early intervention across all complication domains.

### 4.3 Clinical application

The practical utility of this RDR risk model is highlighted by its ease of use and the decision curve analysis. All predictors in Model II are regularly collected in routine diabetes care, making the nomogram readily implementable without special equipment. A clinician or health worker can simply input a patient’s values into the nomogram (or a calculator or web tool based on it) to obtain an individualized risk estimate. This has significant implications for diabetes management and screening: it enables a shift toward risk-based referral for retinal exams. For example, using our model, a general practitioners or endocrinologists could identify a subset of diabetics who have RDR, say, >20% predicted probability of referable DR-those patients would be prioritized for prompt dilated fundus examination by an ophthalmologist. Conversely, patients scoring below a low threshold (perhaps <10% risk) might be safe to defer immediate specialist referral and continue periodic monitoring, especially in resource-constrained settings. By applying such a strategy, healthcare systems could optimize resource allocation, focusing ophthalmologic services on those most likely to benefit and reducing unnecessary referrals of low-risk individuals. The decision curve analysis confirmed that using the nomogram for referral decisions would confer net benefit across a broad range of threshold choices (from very sensitive to more specific criteria). This flexibility means the tool can adapt to different clinical policies: whether one prefers to “catch” as many cases as possible (lower threshold) or avoid false positives (higher threshold), the model adds value over no model at all. An illustrative scenario is a moderate threshold of 20–30% risk: at that level, the DCA suggests substantial clinical benefit in terms of earlier DR detection and prevention of vision loss. Thus, the nomogram can be tailored to the context – for instance, a community screening program might use a low cutoff to maximize sensitivity, whereas a specialist clinic triaging referrals might use a higher cutoff to ensure specificity. In all cases, the individualized risk estimate fosters a more nuanced approach than one-size-fits-all screening. Moreover, because the model is simple, it could be integrated into electronic health record systems to automatically flag high-risk patients during routine visits (e.g., via an embedded risk score or alert). This kind of integration would make risk stratification seamless and could prompt clinicians in real time to arrange retinal examinations for those flagged at high risk. From a patient education standpoint, the nomogram could be a useful counseling tool. For example, showing a patient how their risk score would drop with a lower HbA1c or blood pressure (nomogram scenarios) might motivate improved adherence to therapy. In essence, our model encapsulates the multifactorial nature of DR risk into a tangible score, which can help communicate risk to patients and drive home the importance of holistic risk factor control. Overall, the real-world application of Model II is as a decision support tool that bridges the gap between primary diabetes care and specialty ophthalmology, ensuring that each patient’s need for retinopathy screening is assessed on the basis of objective risk.

### 4.4 Limitations and future directions

Despite the strengths of our study, several limitations warrant consideration. First, our nomogram was developed and validated using cross-sectional datasets derived from community-based screenings in Northwest China. This design limits the model’s ability to predict future incidence of RDR, as it captures only a single time-point diagnosis rather than disease progression over time. Furthermore, voluntary participation in these screenings may introduce selection bias, as participants may differ from the broader diabetic population in health behavior, socioeconomic status, or access to care. As such, the generalizability of our findings to other regions, ethnicities, or healthcare systems may be limited. Future prospective, multicenter studies across geographically and demographically diverse populations are essential to improve external validity and ensure broader applicability.

Second, the model primarily incorporates static clinical variables, whereas diabetes is a dynamic condition influenced by time-varying factors such as glycemic variability, blood pressure fluctuations, and renal function changes. Incorporating longitudinal data—such as visit-to-visit HbA1c trajectories, systolic blood pressure variability, or progressive microalbuminuria—may enhance predictive granularity and support the development of temporally adaptive risk models. Such refinements would be particularly valuable for identifying patients at imminent risk of DR progression, enabling more timely interventions.

Third, the absence of certain potentially important predictors—such as dietary habits, physical activity, family history, and genetic markers—may reduce the comprehensiveness of our risk stratification approach. Integrating these variables in future studies could improve the personalization and precision of DR risk prediction, particularly in subgroups with atypical risk profiles.

Fourth, while our model was constructed using logistic regression for its interpretability and ease of implementation, advanced machine learning (ML) methods, including gradient boosting, support vector machines, and deep neural networks, have shown superior predictive performance in some DR studies. Nevertheless, ML models often lack transparency, are resource-intensive, and may pose barriers to routine clinical deployment. Head-to-head comparisons of conventional versus ML-based models—focusing not only on accuracy but also on interpretability, clinical utility, and integration feasibility—will be critical for guiding model selection in real-world practice.

Finally, translating prediction models into clinical benefit requires seamless integration with healthcare systems. Embedding the nomogram within electronic health record (EHR) platforms would enable real-time risk assessment by automatically extracting and updating relevant patient data (e.g., HbA1c, blood pressure, renal indices). Such integration could generate dynamic alerts or risk scores within clinical workflows, facilitating timely referral of high-risk patients and supporting targeted screening strategies. In resource-constrained environments, this approach could significantly optimize allocation of ophthalmologic services and reduce preventable vision loss.

In conclusion, future efforts should focus on prospective validation, incorporation of time-dependent and novel predictors, comparative evaluation of modeling strategies, and EHR-based implementation. These advances will further improve the nomogram’s predictive precision, clinical relevance, and scalability, thereby enhancing its role in personalized, risk-based diabetic retinopathy screening and management.

## 5 Conclusion

In conclusion, we have developed a clinically useful and interpretable nomogram for referable DR risk that relies on seven routine variables reflecting glycemic control, blood pressure, and kidney health. Model II offers moderate but consistent accuracy in identifying patients with sight-threatening retinopathy, as evidenced by internal and external validations. The model’s strength lies in its simplicity and strong grounding in pathophysiology, it leverages the interrelated risk factors that drive microvascular damage in diabetes. For clinicians, this tool provides a quick risk assessment that can support decision-making on patient referrals for ophthalmologic evaluation. For patients and health systems, its use could mean earlier detection of vision-threatening DR and more efficient use of specialist resources. The broad threshold range yielding net benefit indicates that the nomogram is robust across various clinical risk tolerances. Future refinements, including prospective studies and integration of dynamic risk factors, could further enhance its performance. Nonetheless, even in its current form, this risk score represents an innovative step toward personalized DR screening, helping bridge the gap between primary diabetes care and preventive ophthalmology. By identifying high-risk individuals before irreversible eye damage occurs, such a model can contribute to reducing the burden of diabetic blindness and improving outcomes through timely intervention.

## Data Availability

The original contributions presented in the study are included in the article/**Supplementary Material**. Further inquiries can be directed to the corresponding authors.
