# Supplementary material for: Universal nomogram for predicting referable diabetic retinopathy: a validated model for community and ophthalmic outpatient populations using easily accessible indicators
Source: Front Endocrinol (Lausanne). 2025 Jun 12;16:1557166. doi: 10.3389/fendo.2025.1557166 (PMC12197942; doi:10.3389/fendo.2025.1557166)
Supplement: Supplementary file 1 [file Table1.docx]

**Table S1** The detailed DR stage in three datasets

| Data sets | ALL | RDR | % of RDR | The detailed stage of RDR | No. | proportion |
| --- | --- | --- | --- | --- | --- | --- |
| The training set  and the test set | 1830 | 362 | 19.78% | Moderate NPDR | 182 | 50.28% |
|  |  |  |  | Severe NPDR and/or DME | 174 | 48.06% |
|  |  |  |  | PDR | 6 | 1.66% |
| The external set | 123 | 98 | 79.67% | Moderate NPDR | 18 | 18.37% |
|  |  |  |  | Severe NPDR and/or DME | 28 | 28.57% |
|  |  |  |  | PDR | 48 | 48.98% |

**TABLE S2** The indicators of model I

| Predictor | Estimate | SE | Z | p | Odds Ratio | β | Lower | Upper |
| --- | --- | --- | --- | --- | --- | --- | --- | --- |
| (Intercept) | -8.072 | 1.161 | -6.955 | 0.0 | 0.0 |  | 0.0 | 0.003 |
| eGFR | -0.002 | 0.004 | -0.449 | 0.654 | 0.998 | -0.002 | 0.988 | 1.003 |
| mALB | 0.0 | 0.0 | 2.258 | 0.024 | 1.0 | 0.000 | 1.0 | 1.001 |
| duration | 0.05 | 0.01 | 5.102 | 0.0 | 1.051 | 0.050 | 1.032 | 1.072 |
| SBP | 0.012 | 0.004 | 3.486 | 0.0 | 1.012 | 0.012 | 1.005 | 1.019 |
| NLR | 0.064 | 0.053 | 1.215 | 0.224 | 1.066 | 0.064 | 0.959 | 1.184 |
| Age | 0.021 | 0.009 | 2.361 | 0.018 | 1.021 | 0.021 | 1.004 | 1.04 |
| SCr | 0.001 | 0.005 | 0.26 | 0.795 | 1.001 | 0.001 | 0.991 | 1.011 |
| BUN | 0.173 | 0.049 | 3.541 | 0.0 | 1.189 | 0.173 | 1.08 | 1.309 |
| HbA1c | 0.222 | 0.048 | 4.576 | 0.0 | 1.248 | 0.222 | 1.135 | 1.373 |
| GLU1 | 0.317 | 0.178 | 1.779 | 0.075 | 1.373 | 0.317 | 0.966 | 1.944 |

| Features | N | AUC | Sensitivity | Specificity | Youden  index | cut-off |
| --- | --- | --- | --- | --- | --- | --- |
| HbA1c | 1830 | 0.666 | 0.671 | 0.601 | 0.273 | 7.355 |
| GLU | 1830 | 0.621 | 0.483 | 0.759 | 0.242 | 1 |
| BUN | 1830 | 0.622 | 0.566 | 0.634 | 0.2 | 5.48 |
| SCr | 1830 | 0.591 | 0.224 | 0.917 | 0.141 | 89.8 |
| Age | 1830 | 0.526 | 0.644 | 0.418 | 0.062 | 68.181 |
| eGFR | 1830 | 0.596 | 0.811 | 0.351 | 0.162 | 72.166 |
| NLR | 1830 | 0.545 | 0.367 | 0.738 | 0.105 | 2.463 |
| SBP | 1830 | 0.574 | 0.472 | 0.653 | 0.126 | 145.62 |
| duration | 1830 | 0.694 | 0.674 | 0.668 | 0.342 | 9.04 |
| mALB | 1830 | 0.616 | 0.42 | 0.772 | 0.192 | 66.04 |

**TABLE S3** The indicators of model II

| Predictor | Estimate | SE | Z | p | Odds Ratio | β | Lower | Upper |
| --- | --- | --- | --- | --- | --- | --- | --- | --- |
| (Intercept) | -6.712 | 0.693 | -9.689 | 0.0 | 0.001 |  | 0.0 | 0.005 |
| SCr | 0.003 | 0.004 | 0.776 | 0.438 | 1.003 | 0.003 | 0.995 | 1.012 |
| BUN | 0.167 | 0.048 | 3.444 | 0.001 | 1.182 | 0.167 | 1.075 | 1.3 |
| HbA1c | 0.222 | 0.048 | 4.646 | 0.0 | 1.249 | 0.222 | 1.137 | 1.372 |
| mALB | 0.0 | 0.0 | 2.186 | 0.029 | 1.0 | 0.000 | 1.0 | 1.001 |
| duration | 0.049 | 0.01 | 5.031 | 0.0 | 1.05 | 0.049 | 1.03 | 1.071 |
| SBP | 0.012 | 0.003 | 3.527 | 0.0 | 1.012 | 0.012 | 1.005 | 1.019 |
| GLU1 | 0.338 | 0.175 | 1.928 | 0.054 | 1.403 | 0.339 | 0.992 | 1.975 |

| Features | N | AUC | Sensitivity | Specificity | Youden  index | cut-off |
| --- | --- | --- | --- | --- | --- | --- |
| HbA1c | 1830 | 0.666 | 0.671 | 0.601 | 0.273 | 7.355 |
| GLU | 1830 | 0.621 | 0.483 | 0.759 | 0.242 | 1 |
| BUN | 1830 | 0.622 | 0.566 | 0.634 | 0.2 | 5.48 |
| SCr | 1830 | 0.591 | 0.224 | 0.917 | 0.141 | 89.8 |
| SBP | 1830 | 0.574 | 0.472 | 0.653 | 0.126 | 146 |
| duration | 1830 | 0.694 | 0.674 | 0.668 | 0.342 | 9.04 |
| mALB | 1830 | 0.616 | 0.42 | 0.772 | 0.192 | 66.04 |

**Table S4** The points and predictive probabilities of model II for nRDR and RDR in the training set and the test set

| **Variable** | **group** | | | ***P*-value***^2^* |
| --- | --- | --- | --- | --- |
|  | **Overall**  N = 1,830*^1^* | **nRDR**  N = 1,468*^1^* | **RDR**  N = 362*^1^* |  |
| **points**  Median (IQR) | 53.412  (45.424 – 64.543) | 51.369  (44.491 – 60.429) | 65.703  (53.598 – 77.477) | <0.001 |
| **predictive probability**  Median (IQR) | 0.150  (0.100 – 0.253) | 0.136  (0.095 – 0.211) | 0.266  (0.152 – 0.420) | <0.001 |

^1^Median (IQR) or Frequency (%);^2^ Wilcoxon rank sum test

**Table S5** The points and predictive probabilities of model II for nRDR and RDR in the external set

| **Variable** | **group** | | | ***P*-value***^2^* |
| --- | --- | --- | --- | --- |
|  | **Overall**  N = 123*^1^* | **nRDR**  N = 27*^1^* | **RDR**  N = 96*^1^* |  |
| **points**  Median (IQR) | 63.078  (52.019 – 81.132) | 51.513  (47.938 – 60.033) | 66.812  (57.492 – 83.222) | <0.001 |
| **predictive probability**  Median (IQR) | 0.240 (0.142 – 0.476) | 0.139 (0.115 – 0.209) | 0.282 (0.186 – 0.506) | <0.001 |

^1^ Median (IQR) or Frequency (%); ^2^ Wilcoxon rank sum test.

**Table S6** The points and predictive probabilities of model II for the stages of RDR in the training set and the test set

| **Variable** | **group** | | | | ***P*-value***^2^* |
| --- | --- | --- | --- | --- | --- |
|  | **Overall**  N = 362*^1^* | **moderate NPDR**  N = 180*^1^* | **severe NPDR or DME**  N = 177*^1^* | **PDR**  N = 5*^1^* |  |
| **points**  Median (IQR) | 65.703  (53.598–77.477) | 62.816  (51.664–74.042) | 68.968  (56.260–83.917) | 65.057  (56.544–82.567) | 0.006 |
| **predictive probability**  Median (IQR) | 0.266  (0.152 – 0.420) | 0.235  (0.138 – 0.372) | 0.305  (0.173 – 0.513) | 0.259  (0.175 – 0.494) | 0.006 |

^1^Median (IQR) or Frequency (%); ^2^ Kruskal-Wallis rank sum test.

**Table S7** The points and predictive probabilities of model II for the stages of RDR in the external set

| **Variable** | **group** | | | | ***P*-value***^2^* |
| --- | --- | --- | --- | --- | --- |
|  | **Overall**  N = 101*^1^* | **PDR**  N = 48*^1^* | **moderate NPDR**  N = 20*^1^* | **severe NPDR or DME**  N = 33*^1^* |  |
| **points**  Median (IQR) | 65.589  (56.163 – 83.235) | 81.579  (74.555 – 92.254) | 48.367  (42.320 – 53.097) | 61.104  (56.163 – 64.778) | <0.001 |
| **predictive probability**  Median (IQR) | 0.268  (0.174 – 0.507) | 0.482  (0.383 – 0.635) | 0.118  (0.086 – 0.150) | 0.220  (0.174 – 0.259) | <0.001 |

^1^ Median (IQR) or Frequency (%); ^2^Kruskal-Wallis rank sum test.

**Table S8** The points and predictive probabilities of the RDR stages of model II in the datasets

| **Variable** | **group** | | | | ***P*-value***^2^* |
| --- | --- | --- | --- | --- | --- |
|  | **Overall**  N = 463*^1^* | **Train**  N = 290*^1^* | **Test**  N = 72*^1^* | **Validation**  N = 101*^1^* |  |
| **points**  Median (IQR) | 65.681  (53.882 – 79.238) | 65.592  (53.231 – 77.373) | 66.264  (56.967 – 78.158) | 65.589  (56.163 – 83.235) | 0.319 |
| **predictive probability**  Median (IQR) | 0.267  (0.154 – 0.445) | 0.265  (0.149 – 0.418) | 0.273  (0.179 – 0.429) | 0.268  (0.174 – 0.507) | 0.272 |
| **type** |  |  |  |  | <0.001 |
| moderate NPDR | 200 (43) | 142 (49) | 38 (53) | 20 (20) |  |
| severe NPDR or DME | 210 (45) | 143 (49) | 34 (47) | 33 (33) |  |
| PDR | 53 (11) | 5 (1.7) | 0 (0) | 48 (48) |  |

^1^ Median (IQR) or Frequency (%); ^2^ Kruskal-Wallis rank sum test; Pearson’s Chi-squared test.
